# Specific Effect of Trace Metals on Marine Heterotrophic Microbial Activity and Diversity: Key Role of Iron and Zinc and Hydrocarbon-Degrading Bacteria

**DOI:** 10.3389/fmicb.2018.03190

**Published:** 2018-12-19

**Authors:** Federico Baltar, Andrés Gutiérrez-Rodríguez, Moana Meyer, Isadora Skudelny, Sylvia Sander, Blair Thomson, Scott Nodder, Rob Middag, Sergio E. Morales

**Affiliations:** ^1^Department of Limnology and Bio-Oceanography, Center of Functional Ecology, University of Vienna, Vienna, Austria; ^2^Department of Marine Science, University of Otago, Dunedin, New Zealand; ^3^National Institute of Water and Atmospheric Research (NIWA)/University of Otago Research Centre for Oceanography, University of Otago, Dunedin, New Zealand; ^4^National Institute of Water and Atmospheric Research, Wellington, New Zealand; ^5^Environment Laboratories, Department of Nuclear Sciences and Applications, International Atomic Energy Agency (IAEA), Monaco, Monaco; ^6^Department of Ocean Systems, Royal Netherlands Institute for Sea Research, Yerseke, Netherlands; ^7^Department of Microbiology and Immunology, Otago School of Biomedical Sciences, University of Otago, Dunedin, New Zealand

**Keywords:** heterotrophic bacterioplankton, trace metals, iron, hydrocarbon-degrading bacteria, bacterioplankton diversity

## Abstract

Marine microbes are an important control on the biogeochemical cycling of trace metals, but simultaneously, these metals can control the growth of microorganisms and the cycling of major nutrients like C and N. However, studies on the response/limitation of microorganisms to trace metals have traditionally focused on the response of autotrophic phytoplankton to Fe fertilization. Few reports are available on the response of heterotrophic prokaryotes to Fe, and even less to other biogeochemically relevant metals. We performed the first study coupling dark incubations with next generation sequencing to specifically target the functional and phylogenetic response of heterotrophic prokaryotes to Fe enrichment. Furthermore, we also studied their response to Co, Mn, Ni, Zn, Cu (individually and mixed), using surface and deep samples from either coastal or open-ocean waters. Heterotrophic prokaryotic activity was stimulated by Fe in surface open–ocean, as well as in coastal, and deep open-ocean waters (where Zn also stimulated). The most susceptible populations to trace metals additions were uncultured bacteria (e.g., SAR324, SAR406, NS9, and DEV007). Interestingly, hydrocarbon-degrading bacteria (e.g., *Thalassolituus, Marinobacter*, and *Oleibacter*) benefited the most from metal addition across all waters (regions/depths) revealing a predominant role in the cycling of metals and organic matter in the ocean.

## Introduction

Microbes mediate the major redox reactions responsible for transforming energy and matter in the world through enzymatic activity (Falkowski et al., 2008). Many of those key enzymes contain or depend on metals, explaining why those metals are essential for life in the ocean (Morel and Price, 2003). Due to their low solubility, metal concentrations are low in the ocean, and the availability of most metals declines rapidly within short distances of the coast (Johnson et al., 1997). Consequently, planktonic microbes are a key control of the biogeochemical cycling of most marine bio-essential metals but simultaneously, these metals control in part the growth of the microorganisms and their cycling of major nutrients like C and N (Morel and Price, 2003).

The best example of this link is the role of Fe as a limiting factor in the ocean, as shown by incubation assays and mesoscale fertilization experiments [see review by (Boyd et al., 2007)] and naturally Fe-fertilized regions (Blain et al., 2007; Pollard et al., 2009). Most Fe, and trace metal, fertilization studies were performed in surface open-ocean waters under light conditions, focusing on the stimulatory response of autotrophic phytoplankton. However, phytoplankton primary production is tightly linked to heterotrophic prokaryotic activity since around half of that production is remineralized through heterotrophic prokaryotes (Ducklow, 2000). These heterotrophs are also competitors for limiting micronutrients such as Fe (Tortell et al., 1996; Maldonado and Price, 1999; Schmidt and Hutchins, 1999; Boyd et al., 2012). Despite this recognized tight link between primary production and heterotrophic prokaryotic activity, few investigations (see below) have tried to study the specific response of heterotrophic prokaryotes to Fe (as opposed to ‘phytoplankton mediated’ response), and even less to the other important trace metals.

The response of marine heterotrophic prokaryotic communities to Fe enrichment differs among studies [see (Obernosterer et al., 2015) for an overview]. For instance, relatively low prokaryotic abundance and heterotrophic production have been reported under iron fertilization experiments (Cochlan, 2001; Hall and Safi, 2001; Oliver et al., 2004; Suzuki et al., 2005; Jain et al., 2015), with no significant changes in bacterial community composition in the Pacific and the Southern Ocean, or the California coastal upwelling (Cary, 2001; Hutchins et al., 2001; Arrieta et al., 2004; Jain et al., 2015). In contrast, increases in prokaryotic abundance and production (Blain et al., 2007; Obernosterer et al., 2008a) as well as changes in microbial community composition (West et al., 2008) have been observed during a phytoplankton bloom induced by natural iron fertilization in the Kerguelen Plateau, and following an *in situ* iron enrichment experiment in the Southern Ocean (Thiele et al., 2012; Singh et al., 2015). These previous studies used molecular tools that do not fully detect the whole bacterial community diversity [i.e., Denaturing Gradient Gel Electrophoresis (DGGE), Catalyzed reporter deposition Fluorescence *In Situ* Hybridization (CARD-FISH), Terminal restriction fragment length polymorphism (T-RFLP)] (Cary, 2001; Hutchins et al., 2001; Arrieta et al., 2004; West et al., 2008). Even when high throughput approaches were used (Thiele et al., 2012; Singh et al., 2015), studies were performed under natural or artificial light:dark cycles, which can confound the response of specific heterotrophic prokaryotes to the trace metals (due to the indirect stimulatory effect on heterotrophs via the increase in organic matter produced by autotrophs). Thus, it is not surprising that when shifts in community composition have been reported they basically highlight a decrease in alpha-diversity due to the growth of copiotrophic phylogenetic groups that usually respond to phytoplankton blooms like Roseobacter, Gammaproteobacteria, and Cytophaga-Flavobacterium (Kataoka et al., 2009; Jamieson et al., 2012; Thiele et al., 2012; Singh et al., 2015).

To specifically study the preference and/or susceptibility of heterotrophic bacterioplankton to trace metals and reveal the potential key bacterioplankton competitors reacting to trace metals *per se* it is necessary to bypass the effect of photoautotrophically generated organic matter by performing dark experiments. A limitation to bear in mind in this study is that the dark conditions will ‘short-cut’ the phytoplankton-bacteria coupling in the photic zone, however, at the same time, provide new insights on bacterial response to Fe/metal availability, otherwise hidden by phytoplankton-mediated response. Moreover, this (dark) experimental strategy is particularly relevant for heterotrophic activity ongoing below the euphotic zone. Thus, this approach simplifies partitioning response to metal addition by limiting the role of photoautotrophs, and allowing new insights into the preferences of heterotrophs. Previous dark incubation experiments showed increases in heterotrophic prokaryotic activity in response to Fe addition when supplemented together with carbon using marine planktonic communities from the Southern Ocean (Church et al., 2000), and from the Kerguelen Islands (Obernosterer et al., 2015), while Fe single addition appear to stimulate production only in naturally enriched waters around the Kerguelen Islands. The only available study specifically reporting on changes in heterotrophic community composition in response to trace metals by using light and dark incubations, utilized DGGE but not next generation sequencing (Jain et al., 2015). Still, these authors found a contrasting relative decrease in *Rhodospirillales* in the dark relative to the light:dark cycle incubations, which further supports a strong link between phytoplankton bloom and *Rhodospirillales* under light incubation (Jain et al., 2015). Together, these results indicate contrasting patterns in heterotrophic prokaryotic community composition that can be obtained when trace metal enrichment experiments are incubated under light versus dark conditions. However, fingerprinting techniques like DGGE may fall short of providing a comprehensive picture of the bacterial response to trace metals.

Here we combined next generation amplicon sequencing with dark incubations and trace metal clean sampling and sample handling techniques to unravel the response of heterotrophic bacterioplankton abundance, activity and diversity to Fe. Since metals other than Fe are known to be important for heterotrophic prokaryotes (Morel et al., 2003), we also tested the response of heterotrophic prokaryotes to another five trace metals important in the biogeochemistry of the ocean (Co, Mn, Ni, Zn, and Cu) individually and in a combination treatment containing all metals together. Furthermore, to gain a more comprehensive picture of the potential role of trace metals in the marine microbial carbon cycle, we performed experiments using both surface and deep (down to 500 m) waters, from coastal and open-ocean waters. With this, we found a stimulation of prokaryotic heterotrophic activity by trace metals like Fe (surface and deep waters) and Zn (deep waters). Finally, we identified the main specific heterotrophic prokaryotic taxa responding (increasing/decreasing) to different trace metals, and thereby identifying the main sensitive and the most competitive heterotrophic prokaryotes against other members of the community like phytoplankton, revealing a key role of hydrocarbon-degrading bacteria.

## Materials and Methods

### Study Site, Regional Oceanographic Settings and Experimental Setup

**FIGURE 1:**
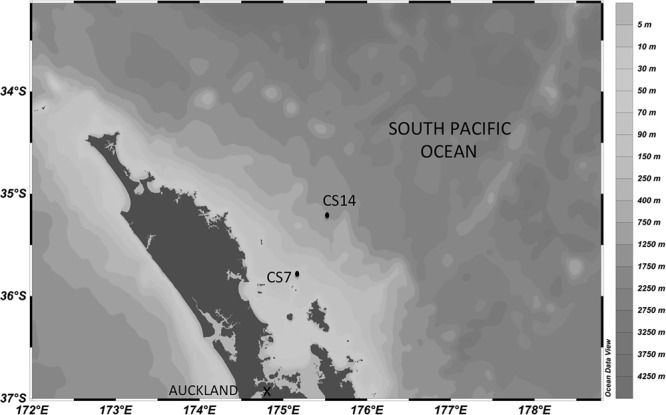
Map depicting the location of the stations were the water for the experiments was collected.

To study the response of contrasting bacterioplankton communities to different trace metals, seawater was collected from a coastal (shelf) and an offshore (slope) station offshore New Zealand (Figure 1), from shallow (20 m) and deep (100–500 m) waters, and supplemented with different trace metals. The deep sample at the slope station was collected at 500 m, whereas we selected 100 m as our deep shelf sample because the water column depth at the shelf station was ca.150 m (Table 1).

**Table 1:** Physicochemical and biological properties of the seawater collected for the trace metal addition experiments, ordered by rows starting with the shelf-surface, followed by the shelf-deep, slope-surface and finally the slope-deep experiment.

Station (location)	Depth (m)	Date	Long (°E)	Lat (°N)	Temp (°C)	Salinity (PSS-78)	Oxygen (mmol/ kg)	Fluor (mg/ m3)	Chla (mg/m3)	PO_4_^3^^-^ (μM)	NH_4_ (μM)	NO_3_-N (μM)	Si(OH)_4_ (μM)	PON (mg/m3)	POC (mg/m3)	PA (cells/ml)	%HNA	PHP (pmol Leu/l/h)
CS07 (shelf)	20	20/05/ 2016	175.17	–35.78	19.97	35.62	212.32	0.63	0.35	<0.03	0.72	0.32	0.10	9.4	31.7	3.2E+05	33.4	136.7
CS07 (shelf)	100	20/05/ 2016	175.17	–35.78	17.39	35.42	198.43	0.25	0.09	1.12	0.69	17.21	1.00	2.1	12.7	1.8E+05	36.6	83.1
CS14 (slope)	20	19/05/ 2016	175.51	–35.21	20.79	35.71	210.42	0.18	0.12	<0.03	1.19	0.51	0.28	5.5	32.2	3.8E+05	32.3	115.3
CS14 (slope)	500	19/05/ 2016	175.51	–35.21	10.09	34.77	191.23	0.06	0	0.40	0.71	6.31	0.91	2.4	8.9	1.6E+05	35.0	51.5

*Lat, latitude; Long, longitude; Temp, temperature; Fluor, fluorescence; Chla, chlorophyll a; *NO*_*3*_-N, Nitrate + Nitrite Nitrogen; *PON*, particulate organic nitrogen; *POC*, particulate organic carbon; *PA*, prokaryotic abundance; *%HNA*, percentage of high nucleic acid content cells; PHP, prokaryotic heterotrophic production*.

The seawater samples were collected from the inner edge of the East Auckland Current (EAUC). The EAUC extends to at least 2000 m water depth (Sutton and Chereskin, 2002) and is a highly variable current system, with re-circulation in several eddy structures (Roemmich and Sutton, 1998). Due to the narrow width of the continental shelf along the northeast coast of the North Island (<40 km-wide), offshore flows from the EAUC periodically encroach onto the continental shelf across the shelf-break under favorable wind conditions (Sharples, 1997). Wind-forced upwelling may occur in early spring to early summer on the inner to mid-shelf, with seasonal downwelling into late summer causing strongly stratified conditions on the shelf, with interactions caused by the proximity of the EAUC apparent on the outer shelf-upper slope (Proctor and Greig, 1989; Sharples and Greig, 1998; Zeldis et al., 2004; Longdill et al., 2008). These event-driven processes have profound implications for the supply of nutrients onto the shelf (Sharples, 1997) and for driving the marine ecosystem from autotrophy to heterotrophy from spring to summer (Zeldis, 2004). In addition, internal waves also lead to the injection of nutrients from deeper water into the surface ocean, as documented north of the Poor Knights Islands on the northeast North Island coast (Sharples et al., 2001).

The seawater samples collected were characterized in terms of physicochemical and biological parameters (Table 1), and used to setup incubations. A wide variety of treatments were prepared, which included individual additions of Fe, Co, Mn, Ni, Zn, Cu and a combination of all those metals at the same concentrations into a combined treatment (called “Mix”). This selection included trace metals of different distribution types (Bruland and Lohan, 2006): scavenged (Mn), nutrient (Ni, Zn), and hybrid (Fe, Co, and Cu). All the treatments and the unamended controls were prepared in 1 L triplicate HDPE bottles that were acid cleaned with HCl following GEOTRACES guidelines. To keep the results as realistic as possible, the concentration of trace metals added to the treatment was calculated from data obtained from the same stations and depths 1 year prior this cruise. We used that data to calculate 5 times the maximum observed concentration of each of those metals, which resulted in final concentrations of 0.12 nM for Co, 6 nM for Mn, 7 nM for Cu, 12 nM for Fe, 18 nM for Ni, and 20 nM for Zn. The combined ‘Mix’ treatment had all metals added in the same concentrations as used in individual metal treatments. Bottles were incubated in the dark at *in situ* temperature for 4 days. At the end of incubations samples were collected for cell counts, prokaryotic heterotrophic production, and community composition (based on the 16S rRNA gene) the triplicates of each treatment and controls, as described below.

Seawater was collected using an autonomous trace metal clean rosette equipment with GO-FLO samplers (General Oceanics). GO-FLO samplers were taken of the rosette and subsampled inside the NIWA/University of Otago mobile trace metal lab. All sampling manipulations were performed under trace metal clean conditions inside the mobile clean lab under a HEPA laminar flow hood (ISO class 5). The solutions used for the additions of the metals were dilutions of 1000 ppm ICP-MS standards (High-Purity Standards) and were made before the expedition in 0.07 M quartz distilled HCl to keep the metals in solution. Subsequent additions of these solutions to seawater led to a final HCl concentration in the incubations between 2 and 24 μM in the individual metal additions and 70 μM in the mix addition which did not have a discernible effect on the pH of the incubated seawater. Given that the IPM-MS standards are made up in 2% nitric acid, the metal additions lead to concurrent nitrate additions (Supplementary Table S2). However, the concurrent addition of N (NO_3_) did not seem to have an effect in stimulating bacterioplankton growth. This can be clearly seen, for example, by the fact that even though the Ni and Zn additions had the highest metal concentration added (and thereby also NO_3_ concentrations), there was no effect in the prokaryotic growth in those treatments, except for the addition of Zn to deep water where the NO_3_ concentration we added is insignificant compared to the ambient concentrations. The fact that those treatments with similar amounts of added trace-metals and therefore NO_3_ did not have a similar response in the community composition, and Fe caused the highest impact on production but was not accompanied by highest NO_3_ levels, suggest that NO_3_ did not seem to affect the production rates nor community composition but the trace metals rather than NO_3_ caused the observed response. This lack of influence of the N addition supplemented with the trace metals is not surprising when a simple back of the envelop calculation is performed: assuming a N quota of 3.28 fg/cell, the N needed to account for the prokaryotic abundance/biomass increase observed in the Mix treatment in the surface-slope experiment [where the biggest prokaryotic abundance (PA) change occurred], would be around 0.33 μM. This would reduce the N concentration in the surface-slope experiment (which was also the one with the lowest ambient N concentrations) from 1.03 to 0.70 μM N. This concentration would be above what is considered limiting in the sense of triggering a response in bacterial growth due to N limitation. The Mix treatment in the surface-slope experiment was the most extreme case we encountered, and if we move to the second strongest bacterial growth response we observed, the Cu treatment of the surface-slope treatment, the N needed to account for the PA biomass increase during that treatment would only be 0.16 μM.

### Salinity, Dissolved Oxygen, Fluorescence, Chl a, Nutrients and Particulate Organic Matter

Salinity, temperature, dissolved oxygen and fluorescence were measured with a Sea-bird electronics (SBE) 911plus CTD and a 10-L SBE 32 rosette water sampler. Seawater for dissolved inorganic nutrients (Nitrate + Nitrite, Ammonia, dissolved reactive phosphorus), particulate organic matter (POC and PON), and chlorophyll a (chl-a) concentration, were sampled from the Niskin using acid-washed silicone tubing. Nutrient samples were filtered through Whatman GF/F filters into clean 250 mL polyethylene bottles and kept at -20°C until analysis using an Astoria Pacific API 300 micro-segmented flow analyser (Astoria-Pacific, Clackamas, OR, United States) according to the colorimetric methods described in (Law et al., 2011). POC and PON samples were analyzed using an Elementar Vario EL 111 CHN analyzer (Elementar Analysensysteme GmbH, Hanau, Germany) following standard combustion techniques. Samples for chl-a analysis were filtered on-board on Whatman GF/F filters using low vacuum (<200 mm Hg), filters were folded and placed in 1.5 mL cryovials at -80°C until analysis following 90% acetone extraction and spectrofluorometry standard methods.

### Heterotrophic Prokaryotes Abundance

Abundance of heterotrophic prokaryote assemblages were determined by flow cytometry. Samples (1.6 ml) were preserved with glutaraldehyde (2% final concentration), left 15 min at 4°C in the dark to fix, deep frozen in liquid nitrogen and stored at -80°C until analysis. Once in the lab, fixed samples were thawed, stained in the dark for a few minutes with a DMS-diluted SYTO-13 (Molecular Probes Inc.) stock (10:1) at 2.5 μM final concentration, and run through a BD Accuri^TM^ flow cytometer with a laser emitting at 488 nm. High and Low Nucleic Acid content prokaryotes (HNA, LNA) were identified in bivariate scatter plots of side scatter (SSC-H) versus green fluorescence (FL1-H). Samples were run at low or medium speed until 10.000 events were captured. A suspension of yellow–green 1 μm latex beads (10^5^–10^6^ beads ml^-1^) was added as an internal standard (Polysciences, Inc.).

### Bacterioplankton Heterotrophic Production Estimated by [^3^H] Leucine Incorporation

Bacterioplankton heterotrophic activity was estimated from the incorporation of tritiated leucine using the centrifugation method (Smith and Azam, 1992). ^3^H-Leucine (Perkin-Elmer, specific activity = 169 Ci mmol^-1^) was added at saturating concentration (40 nmol l^-1^) to triplicate 1.2 ml subsamples. Controls were established by adding 120 μl of 50% trichloroacetic acid (TCA) 10 min prior to isotope addition. The microcentrifuge tubes were incubated in the dark at *in situ* temperature for 1 h (Table 1). Incorporation of leucine in the replicated sample was stopped by adding 120 μl ice-cold 50% TCA. Subsequently, the subsamples and the controls were kept at -20°C until centrifugation (at ca. 12000 × *g*) for 20 min, followed by aspiration of the water. Finally, 1 ml of scintillation cocktail was added to the microcentrifuge tubes before determining the incorporated radioactivity after 24 to 48 h on a Tri-Carb^®^Liquid Scintillation Counters scintillation counter (Perkin-Elmer) with quenching correction.

### Prokaryotic Community Composition Response as Measured via 16S rRNA Gene Profiles

Samples for DNA analyses were collected by filtering 0.5–1 L of seawater through a 0.22 μm polycarbonate filter. DNA was extracted separately from each filter using a PowerSoil^®^DNA Isolation Kit (Mo Bio, Carlsbad, CA, United States). The manufacturer’s protocol was modified to use a Geno/Grinder for 2 × 15 s instead of vortexing for 10 min and a final elution of 50 μL solution C6 (sterile elution buffer, 10 mM Tris) was used. DNA concentration was measured using a Nanodrop Spectrophotometer from Thermo Fisher. The median 260/280 ratio was 1.5 with a lower quartile of 1.4 and an upper quartile of 1.7. 16S rRNA gene amplicon sequencing was carried out using the Earth Microbiome Project barcoded primer set and conditions (Caporaso et al., 2012). All amplicons (independent replicates) were run on an Illumina HiSeq 151bp x2 run. QIIME 1.9.1 was used to quality filter sequences to 151 base pairs, using default parameters (Caporaso et al., 2012). Operational taxonomic units (OTUs) were defined by clustering sequences with at least 97% similarity. Open-reference OTU picking was carried out using the SILVA 123 release reference library and UCLUST (Quast et al., 2012). The OTUs were assigned taxonomy using BLAST-based classification and the SILVA reference database. Data was rarefied (subsampled) ten times to a depth of 11,000 sequences per sample before merging of OTU tables. Low sequence depth samples (<11,000 sequences), were removed. Downstream analysis was conducted using the merged biom file. OTU tables were transformed to account for multiple rarefactions by calculating a mean and all data was rounded prior to downstream analysis using the phyloseq package (McMurdie and Holmes, 2013) in R (R Core Team, 2015). All sequences of this study are available under BioProject PRJNA504304.

### Statistical Analyses

To check for significant differences in heterotrophic prokaryotic abundance, production and percentage of high nucleic acid content cells among treatments and controls one-way ANOVAs were used, followed by Tukey-HSD *post hoc* tests to assess the individual significant effects between treatments. Results from the Tukey-HSD were included in the plots in the form of letters, so that all treatments labeled with the same letter were not significantly different (i.e., did not increase or decrease significantly relative to each other). In all analyses, parametric assumptions were first checked using the Shapiro–Wilk test for normality and the Levene’s test for equal variance. All analyses were run with the JMP^®^Pro 10.0.0 Statistical Software (SAS Institute Inc., Cary, NC, United States).

Alpha diversity measures (Shannon and richness) were calculated using the *estimate_richness* command, and significance was tested by means of the non-parametric Kruskal–Wallis test. To identify treatment responsive OTUs the dataset was separated by sampling location (surface slope, deep slope, surface shelf, and deep shelf). For each site each metal treated sample was compared to its corresponding no treatment control. Treatment effects were detected using the *exactTest* function in the edgeR package (Robinson et al., 2010).

## Results

### Biogeochemical Characteristics of the Shelf and Slope Waters

To study the importance of different types of trace metals on heterotrophic bacterioplankton we conducted four addition experiments. To account for potential differences in response of the bacterioplankton communities due to location, these experiments were done with coastal (shelf) and open-ocean (slope) waters off New Zealand (Figure 1), targeting shallow euphotic and deep mesopelagic waters (Table 1). The water column depth at the shelf station was ca. 150 m, so we selected 100 m as our deep shelf sample. In contrast, the deep sample at the slope station was collected at 500 m. Still, both deep water samples were richer in nutrients than shallow waters presenting concentrations >1 order of magnitude higher of phosphate (i.e., <0.03 μM at the shelf-shallow and slope-shallow waters versus 1.12 and 0.40 μM at the shelf-deep and slope-deep, respectively) and nitrate (i.e., 0.32 and 0.51 mg m^3^ at the shelf-shallow and slope-shallow waters versus 17.2 and 6.3 μM at the shelf-deep and slope-deep, respectively). The shelf-shallow waters presented higher chl-a than the slope (i.e., chl-a concentration of 0.63 and 0.25 mg m^3^ at the shelf-shallow and slope-shallow samples, respectively). Although particulate organic carbon (POC) concentrations were similar between the shelf and slope shallow waters, particulate organic nitrogen (PON) were higher in the shelf waters (9.4 and 5.5 mg m^3^ at the shelf-shallow and slope-shallow, respectively), indicating a potentially richer and more labile material at the shelf. Nevertheless, the heterotrophic prokaryotic abundance (3.2 and 3.8 × 10^5^ cells ml^-1^ in the shelf-shallow and slope-shallow waters, respectively) and production (137 and 115 pmol Leu l^-1^ h^-1^) were similar in the shallow shelf and slope waters, indicating weak differences, if any, in heterotrophic bacterioplankton stocks and activities despite the differences observed in chl-a and PON concentrations. As expected, the deep waters in both the shelf and the slope presented lower chl-a, POC and PON concentrations, heterotrophic bacterioplankton cell numbers and activities than the surface waters.

### Response of Heterotrophic Bacterioplankton Stocks and Activities to Different Trace Metals

**FIGURE 2:**
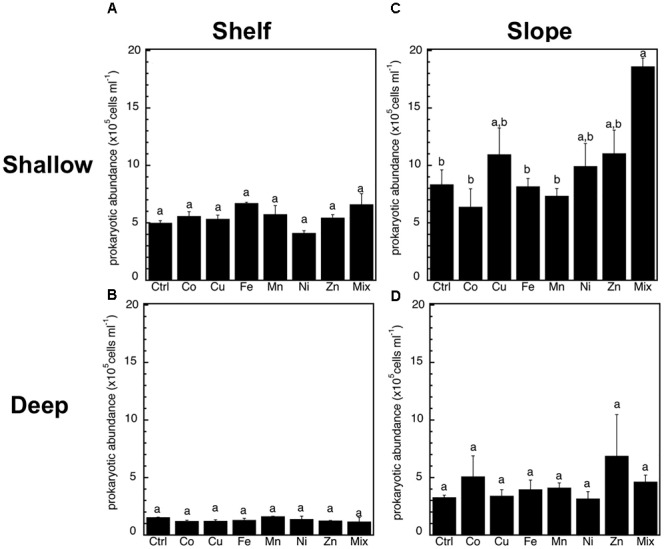
Mean (±SE, *n* = 3) heterotrophic prokaryotic abundance in response to Co, Cu, Fe, Mn, Ni, Zn and the combination of all (Mix), and in unamended controls (Ctrl), experiments performed with water collected from **(A)** surface-coastal, **(B)** deep-coastal **(C)** surface-open ocean, and **(D)** deep-open ocean. Results of Tukey-HSD shown as lowercase letters: levels not connected by same letter are significantly different.

**FIGURE 3:**
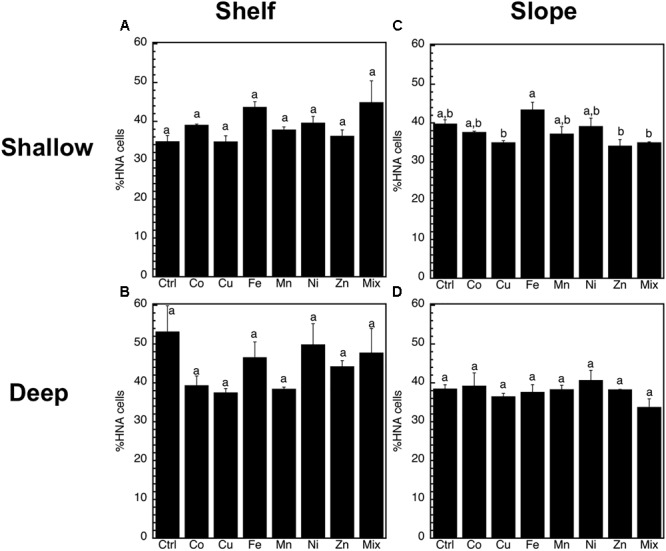
Mean (±SE, *n* = 3) percentage of high nucleic acid content cells (%HNA) in response to Co, Cu, Fe, Mn, Ni, Zn and the combination of all (Mix), and in unamended controls (Ctrl), experiments performed with water collected from **(A)** surface-coastal, **(B)** deep-coastal **(C)** surface-open ocean, and **(D)** deep-open ocean. Results of Tukey-HSD shown as lowercase letters: levels not connected by same letter are significantly different.

**FIGURE 4:**
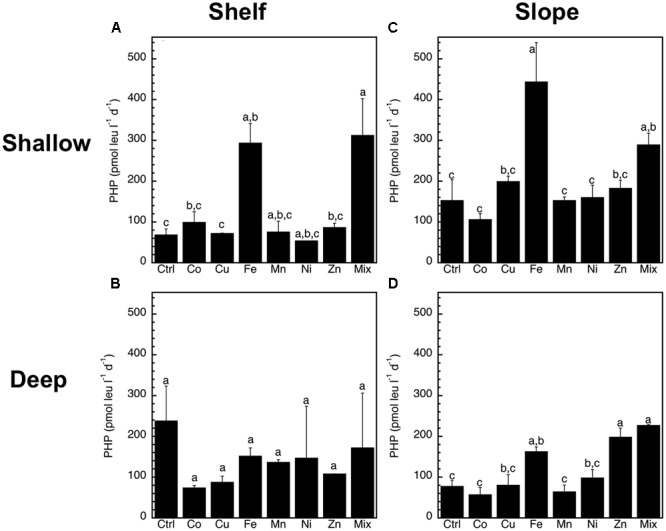
Mean (±SE, *n* = 3) prokaryotic heterotrophic production (PHP) rates (pmol leu l^-1^ d^-1^) in response to Co, Cu, Fe, Mn, Ni, Zn and the combination of all (Mix), and in unamended controls (Ctrl), experiments performed with water collected from **(A)** surface-coastal, **(B)** deep-coastal **(C)** surface-open ocean, and **(D)** deep-open ocean. Results of Tukey-HSD shown as lowercase letters: levels not connected by same letter are significantly different.

The abundance of heterotrophic prokaryotes increased significantly (*p* < 0.05, ANOVA, Tukey HSD) only in response to the Mix treatment of the slope-shallow experiment (Figure 2). No significant increases or decreases due to the trace metals additions were found for any other experiment/treatment. The relative proportion of high nucleic acid cells (%HNA) also presented low levels of responses to the trace metal additions, with no significant (*p* < 0.05, ANOVA, Tukey HSD) differences between the control and any of the treatments (Figure 3). Despite this lack of changes in the abundance of heterotrophic prokaryotes and %HNA, significant increases in the prokaryotic heterotrophic activity were found in response to some trace metals (Fe, Mix, and Zn). Specifically, prokaryotic heterotrophic production rates were significantly (*p* < 0.05, ANOVA, Tukey HSD) increased in response to Fe and Mix in the shelf-shallow (Figure 4A), the slope-shallow (Figure 4C), and slope-deep (Figure 4D) experiments. Besides Fe and Mix, also Zn significantly increased prokaryotic heterotrophic production in the slope-deep experiment (Figure 4D). This increased heterotrophic activity is also consistent with the relatively higher (although not significant) abundance of heterotrophic prokaryotes found in the slope-deep experiment in response to Zn (Figure 2D).

### Prokaryotic Community Composition and Diversity Changes in Response to Trace Metals

Prokaryotic alpha diversity, quantified as richness and Shannon index, remained relatively stable in response to the different metal additions in all the experiments (Supplementary Figures S1, S2 and Supplementary Table S1).

**FIGURE 5:**
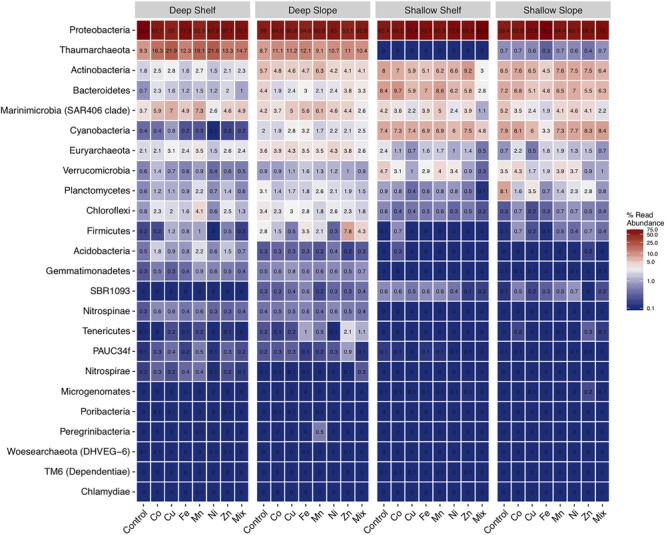
Heatmap of the mean (±SE, *n* = 3) relative abundance of prokaryotic16S rRNA sequences at the phylum level, for experiments performed with water collected from deep-coastal (shelf), deep-open ocean (slope), surface (shallow)-coastal, and surface-open ocean (slope). Only phyla with a relative abundance ≥1% are included.

At the phylum level, in the controls, the most abundant group in all sites and depths was the Proteobacteria, with relative abundances >59% (Figure 5 and Supplementary Figure S3). Thaumarchaeota followed as the second most abundant phylum, but only in the deep samples, with relative abundances of ca. 9% in the controls (but <1% in the surface samples). Actinobacteria, Bacteroidetes, Cyanobacteria, were also more abundant in the surface than in the deep waters with relative abundance of around 3–8%.

The most noticeable change at the phylum level in response to metal treatments was the strong decrease in the relative abundance of Verrucomicrobia in both surface experiments, particularly in Cu, Zn and Mix treatments (Supplementary Figure S3 and Figure 5). This decrease was up to 15-fold in the Mix treatment relative to the control of the shelf-surface experiment. In parallel, Planctomycetes decreased in the shallow-slope experiment from 8% in the control to around 0.7–0.8 in response to Fe and Mix. In contrast, in the shelf-deep experiment, Acidobacteria increased by three–fourfold in response to Co, Mn, and Zn and Firmicutes by up to fivefold in response to Mn and Fe. Also, in the slope-deep experiment, Tenericutes (from 0.2 to 2%) and Firmicutes (from 2.8 to 7.8%) increased in response to Zn.

**FIGURE 6:**
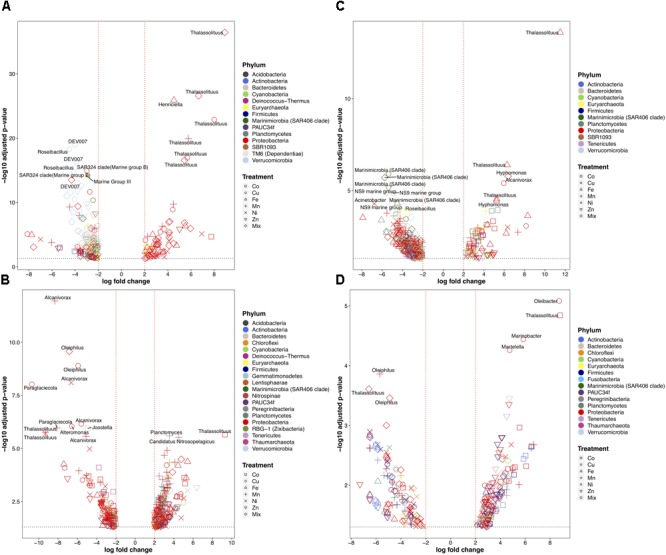
Volcano plots showing only the statistically significant (fold change > 2-fold and *p*-value < 0.05) heterotrophic prokaryotic genera for the experiments performed with water collected from **(A)** surface-coastal, **(B)** deep-coastal **(C)** surface-open ocean, and **(D)** deep-open ocean. *X*-axis indicates the fold change in read abundance when comparing a treated sample to a control. *Y*-axis represents the -log10 transformed *p*-value after removing OTUs with *p*-value < 0.05. Dashed lines (black) represents *p*-value of 0.05 and (red) fold change > 2-fold. Taxonomy represents phylum level classification of affected OTUs. Significance was determined using Fisher’s Exact test against control samples for each treatment (see section “Materials and Methods” for details).

The analyses at the genus level revealed more noticeable differences between the experiments and across treatments (Figure 6 and Supplementary Figure S4). Most of the phyla detected contained genera that increased and/or decreased in response to different metals (Figure 6). At the genus level, Mn, Cu and Mix were the metal treatments causing the strongest decreases in all the surface and deep-water experiments. Interestingly, the most decreased genera in response to our metal experiments were depth-dependent: the most affected genera in the two deep-water experiments genera belonged to Proteobacteria (e.g., *Alcanivorax, Oleiphilus*, and *Thalassolituus*) (Figures 6B,D), but in the two surface-water experiments they belonged to a more diverse group of taxa (mostly uncultured or difficult to culture bacteria like SAR324, SAR406, NS9, DEV007, and *Roseibacillus*) (Figures 6A,C). Although some Proteobacteria genera were reduced in relative abundance in response to metals, most were positively affected by metal additions. The Proteobacterial genus *Thalassolituus* increased the most in relative abundance in response to trace metals in most of the experiments. *Thalassolituus* increased in response to Cu, Mn, and the Mix in the shelf-shallow experiment (Figure 6A); in response to Co in the shelf-deep experiment (Figure 6B); in response to Fe in the slope-shallow experiment (Figure 6C); and in response to Co in the slope-deep experiment (Figure 6D). Other genera that strongly increased in response to metals included the Proteobacteria *Henriciella* (in response to Fe) in the shelf-shallow experiment; *Planctomyces* and Candidatus Nitrosopelagicus (in response to Mn) in the shelf-deep experiment; *Hyphomonas* (in response to Fe and Mix) and *Alcanivorax* (in response to Cu) in the slope-shallow experiment; and the Proteobacteria *Martelella, Marinobacter*, and *Oleibacter* (in response to Cu) in the slope-deep experiment.

## Discussion

### Experimental Considerations: Focusing on Heterotrophic Prokaryotes

The *in situ* biogeochemical characteristics of the seawater collected to perform our experiments revealed very contrasting physicochemical and biological properties (Table 1). As expected, these included more eutrophic waters in coastal than open-ocean waters and also in the dark deep waters than in the sunlit shallow. The low response to trace metal addition in the number of heterotrophic prokaryotic cells and percent of HNA cells, confirms that the concentrations of metals added were within realistic ranges, and that the incubation time was appropriate, since no unrealistic overstimulation was observed. In particular, this is supported by the similarity of the %HNA values in the experiments as compared to the *in situ* waters (Table 1), and the lack of significant changes in the percentage of HNA cells of any of the experiments. This is because higher values of HNA cells are usually associated with growth of heterotrophic prokaryotes in response to high phytoplankton biomass and/or inputs of nutrients or relieve of predatory pressure (Gasol and Giorgio, 2000; Schäfer et al., 2001; Baltar et al., 2010, 2016). Thus the lack of dramatic changes in our experiments indicates a response of the natural community to metal availability, rather than an ‘artefactual’ community developing during the various days incubation (or following marked changes in phytoplankton production and DOM supply).

The lack of response of HNA cells to the trace metal concentrations used not only supports the appropriateness of those concentrations but also suggests a low influence of trace metals on stimulating the HNA cells. Our results contrast with an increase in the percent of HNA cells reported in natural or artificial iron-fertilized patches (Oliver et al., 2004; Obernosterer et al., 2008b). However, those previous studies were based on iron-fertilization *in situ* under natural light conditions that stimulated phytoplankton biomass and production (and thereby increased the percent of HNA cells), whereas our present study was conducted in the dark to focus on the study of the effects of trace metals on heterotrophic prokaryotes. Thus, although trace metal addition experiments performed under light condition can provide very relevant information they might also confound and make it difficult to disentangle the stimulation caused by photoautotroph produced DOM versus the direct effect of trace metals on heterotrophs.

### Trace Metal Additions Have Minimal Impact on the Abundance and Activity of Heterotrophic Prokaryotes Across Multiple Water Types and Depths

The response of marine heterotrophic prokaryotic communities to Fe enrichment differs among studies [see (Obernosterer et al., 2015) for an overview]. However, as explained above, Fe enrichment experiments performed under light conditions do not allow clear testing of direct effects of iron on marine heterotrophic prokaryotes. Using dark incubation to focus on heterotrophic bacteria, a deficiency in Fe has been linked to a reduction in fitness and growth of marine prokaryotes (Kirchman et al., 2000; Fourquez et al., 2014). This is consistent with experimental studies using bacterial strains showing a stimulation of growth in response to Fe addition (Tortell et al., 1996; Fourquez et al., 2014). In a dark incubation experiment in the Southern Ocean, heterotrophic prokaryotic growth was relatively unchanged by additions of Fe alone, but growth increased when Fe was supplemented together with C (glucose) (Church et al., 2000). In another dark incubation experiment of natural microbial communities (off the Kerguelen Islands), single and combined additions of Fe and C stimulated bacterial production at the naturally Fe-fertilized sites, while in high nutrient low chlorophyll (HNLC) waters only combined Fe and C additions caused increased bacterial activities (Obernosterer et al., 2015). However, it is recognized that trace metals other than Fe are also essential for critical enzymes involved in key marine biogeochemical pathways (Morel et al., 2003). An increase in bacterial abundance in response to Fe, Co and the combination of both was observed in an iron fertilization experiment (exposed to light:dark cycles) in the Southern Ocean, suggesting that heterotrophic bacterial growth was limited by Fe, Co or by both (Jain et al., 2015).

In the present study we found minimal changes in the abundance of prokaryotes in response to our trace metal additions. The only significant change was in response to the Mix treatment of the shallow-slope experiments. This finding is consistent with the notion that the shallow-slope (open ocean) waters are those where the strongest limitations of trace metals are expected. This suggests that while Fe alone did not significantly increase the abundance of prokaryotes, the combination of all the mixed metals did. This increase in prokaryotic abundance was consistent with an increase in heterotrophic production, not only in the Mix treatment of the shallow-slope experiment but also in response to Fe. In fact, we found that Fe and Mix where the only treatments consistently increasing the heterotrophic production rates in the 3 out of 4 experiments were significant responses were found (i.e., shallow-shelf, shallow-slope and deep-slope experiments). Only in the deep-shelf experiment were no significant differences observed in response to any metal treatment, consistent with the site being the closest to trace metal inputs (i.e., located in coastal waters and relatively close to the sediments). These results also highlight how heterotrophic prokaryotic activity might be limited mainly, though not exclusively, by Fe in surface open-ocean waters as well as some coastal surface and deep open-ocean waters.

We also found that not only Fe and Mix significantly stimulated prokaryotic heterotrophic production rates, Zn also had a stimulatory effect in the deep-slope experiment. Like phytoplankton, heterotrophic prokaryotes require Fe as electron carriers for respiration in enzymes like aconitase (Fe4S4 active site) and iron rich cytochromes. Different prokaryotic taxa use enzymes specialized for the degradation of particular classes of organic compounds, many of which contain Fe or Zn (a few containing Cu) to perform the additional redox reactions, to the point that the physiological importance of Zn seems to rival that of Fe (Morel et al., 2003). In fact, the number of known Zn-metalloproteins appears to be much larger than that of Fe-metalloproteins (Maret, 2002). The biogeochemical cycle of Zn has recently been shown to be coupled to silicon cycle through the Southern Ocean, although the mechanistic link between the uptake of zinc and silicate by phytoplankton remains unclear (Vance et al., 2017). Maybe the uptake of Zn associated with bacteria growth following phytoplankton growth (and silicate uptake) is related to this coupling. An example of an important enzyme that depend on is alkaline phosphatase (Morel et al., 1994, 2003). Cell-specific alkaline phosphatase has repeatedly been reported to increase toward deep waters (Koike and Nagata, 1997; Hoppe and Ullrich, 1999; Baltar et al., 2009, 2013), which could suggest a relatively stronger need for Zn per cell in deep waters. Whether the limitation of deep offshore prokaryotes by Zn is due to the relative prevalence of alkaline phosphatase or any other Zn-metalloprotein based process requires further research.

### Effect of Iron (and Other Trace Metals) on the Community Composition of Heterotrophic Prokaryotes: Revealing Hydrocarbon-Degrading Bacteria as Key Competitors for Trace Metals

Contrasting results have been previously reported on heterotrophic prokaryotic community composition in response to Fe enrichment (Cary, 2001; Hutchins et al., 2001; Arrieta et al., 2004; West et al., 2008; Thiele et al., 2012; Jain et al., 2015; Singh et al., 2015). However, these previous studies, with the exception of (Thiele et al., 2012) and (Singh et al., 2015), did not use next generation sequencing, and were performed under light:dark cycles. Thus, when shifts in response to Fe were reported, they were related to a decrease in alpha-diversity caused by the stimulation of copiotrophs linked to phytoplankton blooms like Roseobacter, Gammaproteobacteria, and Cytophaga-Flavobacterium (Kataoka et al., 2009; Jamieson et al., 2012; Thiele et al., 2012; Singh et al., 2015). There is one study looking at phylogenetic changes of heterotrophic prokaryotes in response to metals in dark incubations but which also used DGGE instead of next generation sequencing (Jain et al., 2015), precluding an in-depth taxonomic analyses. Still, these authors managed to observe a relative increase in *Rhodospirillales* in the light:dark compared to the dark experiments, supporting the phytoplankton-mediated influence of light in selecting for copiotrophs in metal stimulation experiments.

In the present study we used 16S rRNA gene Illumina sequencing of dark incubations supplemented with Fe (and 5 other metals) to reveal the potential response of specific heterotrophic prokaryotic taxa to different trace metals. We aimed to identify potential competitors against phytoplankton and other heterotrophs for trace metals (bypassing the selective changes that could occur in response to autotrophic growth). We found that although the alpha-diversity remained relatively stable in most treatments/experiments, specific changes were observed already at a broad taxonomic level (phylum) in response to Fe and also to other trace metals. The most noticeable change at the phylum level was the strong decrease up to 15-fold in Verrucomicrobia in both surface experiments (in response to Cu, Fe, Zn, and Mix), and by ca. 10-fold in Planctomycetes in the shallow-slope experiment in response to most metals (although increased by 3.7-fold in response to Mn in the shelf-deep experiment). Those decreases contrasted with the three–fourfold increases of Acidobacteria in response to Co, Mn, and Zn in the shelf-deep experiment, by 10-fold of Tenericutes in the slope-deep experiment, and by ca. three–fivefold of Firmicutes in response to Zn in the slope-deep experiment and to Mn and Fe in the shelf-deep experiment. These results are consistent with the increase in relative abundance of Acidobacteria and decrease in Verrucomicrobia observed in soil bacteria in response to increased Cu concentrations (Naveed et al., 2014; Nunes et al., 2016). Acidobacteria in marine methane-seep sediment have been suggested to be Mn reducers (Beal et al., 2009), which helps explain the observed positive response to Mn. In agreement with our results, increases in response to ZnO in the intestinal microbiota of weaned piglets have been reported for Tenericutes (Yu et al., 2017). Consistent with the positive response of Firmicutes to Fe and Mn in our study, Firmicutes increased after Fe fertilization in the Southern Ocean (Singh et al., 2015), and are together with beta-proteobacteria the model Mn(II)-oxidizing bacteria and among the most abundant bacteria found in soil Fe–Mn nodules (Zhang et al., 2008).

The use of next generation sequencing allowed us to also identify specific genera significantly responding to the different metals. Most of the phyla had genera that increased and/or decreased in response to different metals, highlighting the plasticity or potential of taxa from the same phylum to respond differently to the same or different metals. In doing so, it also confirms the importance of using high taxonomical resolution to discern the effect of trace metals on heterotrophic prokaryotic community composition. Moreover, we found a stronger level of variability in taxonomic shifts compared to heterotrophic activity responses to the different trace metals, which further suggest a high level of functional redundancy, consistent with other aquatic studies (Ofiţeru et al., 2010; Baltar et al., 2012; Aylward et al., 2015; Louca et al., 2016).

The largest losses at the genus level in all experiments were caused by the Mn, Cu, and Mix additions (Figure 6). However, the pattern of response of the top decreasing genera was depth-dependent. In both deep experiments, the top decreased genera belonged to Proteobacteria like *Alcanivorax, Oleiphilus, Thalassolituus*, which are known hydrocarbon-degrading bacteria (Yakimov et al., 2007). In contrast, in the two surface-water experiments, the top decreased genera were SAR324, SAR406, NS9, DEV007, and *Roseibacillus*. These genera are uncultured or very difficult to culture (Orsi et al., 2016), which might indicate why they were preferentially negatively affected by the metal enrichment experiments. SAR324 and SAR406 are considered oligotrophic bacteria (Cho and Giovannoni, 2004), which could also explain an adaptation toward low levels of trace metal concentrations. Consistent with our findings, the Verrucomicrobia DEV007 was recently identified among the most sensitive taxa to multiple metals in a molecular screening of microbial communities for candidate indicators of multiple metal impacts performed in marine sediments from northern Australia (Cornall et al., 2016).

Among the strongest competitors (top increased) for trace metals identified, the case of *Thalassolituus* was noteworthy as this genus repeatedly increased significantly in all experiments; in response to Cu, Mn, Zn, Fe, and Mix in the surface experiments, and to Co in the two deep experiments (Figure 6 and Supplementary Figure S5). Consistent with this, members of *Thalassolituus* have been found on Iron–Manganese Concretions in the Baltic Sea (Yli-Hemminki et al., 2014). These authors also found that in a corresponding experiment, the addition of Fe and oxygen favored the enrichment of *Thalassolituus oleivorans*. This genus is an aerobic heterotrophs known to obligately utilize hydrocarbons (Yakimov et al., 2007).

In the surface water experiments, besides *Thalassolituus*, the other top genera responding to trace metals were *Henriciella* and *Hyphomonas* and *Alcanivorax* (specifically to Fe, Cu, and Mix), which also are hydrocarbon-degrading bacteria. Consistent with the positive response of *Hyphomonas* to Fe and the Mix, members of this genera are reported to be psychrophilic, iron-oxidizing bacteria that are prolific, and ubiquitous members of deep-sea hydrothermal vent communities (Jannasch and Wirsen, 1981), capable of depositing a heavy layer of iron or manganese salts on the cell surface (Moore et al., 1984). *Alcanivorax borkumensis*, is considered the paradigm of obligate hydrocarbonoclastic bacteria and probably the most important representative globally (Yakimov et al., 2007). Members of *Alcanivorax* are siderophore-producing bacteria (Denaro et al., 2014), and its representative genomes encodes genes for the uptake of metals, like magnesium, molybdate, zinc, cobalt, and copper through mgtE, modABC, znuAB- encoded systems, a CorA-like MIT family protein, and Copper-binding protein CopC (Yakimov et al., 2007; Sabirova et al., 2011).

In the deep-water experiments, besides *Thalassolituus*, the other top increasing bacteria in response to the metals were *Planctomyces* and Candidatus Nitrosopelagicus (in response to Mn), and *Martelella, Marinobacter*, and *Oleibacter* (in response to Cu). The increase of *Planctomyces* in response to Mn in our experiment is consistent with some members of *Planctomyces* being Mn-oxidizing bacteria that accumulate iron and/or manganese on the non-cellular stalk (Schmidt et al., 1982), and been found in Fe-Mn-rich hydrothermal mounds in the Juan de Fuca Ridge (Davis et al., 2009). *Oleibacter* is considered a very important obligate hydrocarbonoclastic bacteria in the marine environment (Sanni et al., 2015). The draft genome of the marine rhizobium *Martelella mediterranea* DSM 17316 contains two operons for copper export, indicating resistance for detoxification of copper (Bartling et al., 2017). Members of *Marinobacter* also are known to generate siderophores (Martinez et al., 2000) and to tolerate high concentrations and accumulate copper using its extracellular polymeric substances (EPS) (Bhaskar and Bhosle, 2006).

The fact that most of the top stimulated genera like *Oleibacter, Martelella, Marinobacter, Alcanivorax, Henriciella, Hyphomonas*, and *Thalassolituus*, were hydrocarbon-degrading bacteria, highlights this type of bacteria as key competitors for trace metals, and suggests a strong link between trace metal availability and the degradation of hydrocarbon in the marine environment. In agreement, there are many different metabolic pathways that hydrocarbon bacteria use to degrade hydrocarbons that require metals, like iron-containing oxygenases such as, the alkane monooxygenase, AlkB2, and cytochrome P450 (CYP) (Denaro et al., 2014). These pathways are found in the vast majority of medium to long chain alkane-oxidizing aerobic bacteria (Austin and Groves, 2011). Previous studies have shown that the requirement of Fe increases during the over-expression of the alkane hydroxylase of hydrocarbon degrading bacteria (Staijen et al., 1999), and a reduction in the efficiency of degradation of hydrocarbon under iron limiting conditions (Dinkla et al., 2001). Recent studies on the siderophore-mediated Fe uptake pathway for oil-degrading bacteria, including *Alcanivorax*, revealed that the production of amphiphilic siderophores could be beneficial not only for the solubilization of oil but also for Fe uptake at the cell surface by acting as a biosurfactant for oil emulsification and associating with the cell membrane to prevent siderophore diffusion (Kem et al., 2014). Furthermore, crude oil typically contains metals (Lord, 1991), which could also support a stronger adaptation of hydrocarbon degrading bacteria to a wide range of trace metal types and concentrations.

Nevertheless, our results also suggest that this potential important link between hydrocarbon degrading bacteria and trace metals does not always result in increasing abundances of those bacteria, since in the deep water experiments we also found that many of these hydrocarbon degrading bacteria were also among the most negatively affected by the metals. This may represent different life strategies (fast growers vs. scavengers) of surface versus deep hydrocarbon degrading bacteria, and might indicate fundamental differences in this link (hydrocarbon-degrading bacteria and trace-metals) between surface and deep waters, in particular; and in the response to metals of surface versus deep heterotrophic prokaryotic communities, in general. These depth-related (and site-related) differences could be due to shifts in the relative abundance of specific ecotypes (ribotypes) within a given taxon (e.g., within a same genus). In other words, these differences could be triggered by intrinsic differences among surface and deep ecotypes within a same taxonomic level able to respond differentially to metal additions. In fact, for the same oceanographic station, trace metals that positively stimulated some prokaryotic genera in surface waters were distinct from those that stimulated the same genera in deep samples (e.g., *Thalassolituus* in Figure 6). However, more research is needed to confirm this hypothesis. Also, this potential differentiation between surface and deep-water hydrocarbon degrading bacterial response to trace metals indicates that the phylogenetic conservation level of this investigated trait seems to be shallow. This is consistent with the recent framework suggesting that traits like the ability to use or take up substrates are shallowly conserved, and taxa share these traits only within small, shallow clades (Martiny et al., 2015).

## Conclusion

This is the first study coupling dark incubations with next generation sequencing to specifically target the phylogenetic response of heterotrophic prokaryotes to Fe enrichment. Furthermore, this study also included enrichment of 5 other metals (and the combination of all), and experiments performed at different marine environments (surface versus deep, coastal versus open-ocean waters). As expected, the response observed in heterotrophic prokaryotic activity and diversity was different to previous trace metal enrichment experiments performed under (natural or artificial) light conditions, highlighting the importance of bypassing the influence of phytoplankton growth and organic matter production on heterotrophic prokaryotes to specifically decipher the response of heterotrophic prokaryotes to trace metal additions. We also found evidence of heterotrophic prokaryotic activity being limited or stimulated by Fe not only in surface open-ocean waters but also in surface coastal and deep open-ocean waters, implying a wider influence of Fe not only restricted to sunlit waters. Deep open-ocean heterotrophic activities did increase in response to Zn, also suggesting a potential important role of this metal in deep-ocean biogeochemical cycling. Next generation sequencing allowed us to further reveal specific main taxa susceptible to, or competitors for the different trace metals. The trace metals causing the strongest shifts to heterotrophic prokaryotic genera were Cu, Mn, Zn, Fe, and the Mix treatment, suggesting those as key trace metals affecting heterotrophic prokaryotic community composition. The most susceptible genera to trace metals were hydrocarbon-degrading bacteria in the deep-water experiments, and uncultured bacteria in the surface water experiments. Interestingly, the most beneficiated and presumably best competitor taxa in all the experiments were hydrocarbon-degrading bacteria (particularly in surface waters where the strongest metal limitations are expected), revealing a potential predominant role of these type of bacteria in the cycling of metals and its associated cycling of organic matter in the ocean.

## Author Contributions

FB designed and performed the research and wrote the paper. AG-R, MM, IS, SS, BT, SN, and SM performed the research and edited the paper. RM designed and performed the research and edited the paper.

## Conflict of Interest Statement

The authors declare that the research was conducted in the absence of any commercial or financial relationships that could be construed as a potential conflict of interest. The reviewer KB declared a shared affiliation with no collaboration with one of the authors FB to the handling Editor at the time of review.
