# Supplementary material for: Specific Effect of Trace Metals on Marine Heterotrophic Microbial Activity and Diversity: Key Role of Iron and Zinc and Hydrocarbon-Degrading Bacteria
Source: Front Microbiol. 2018 Dec 19;9:3190. doi: 10.3389/fmicb.2018.03190 (PMC6306045; doi:10.3389/fmicb.2018.03190)
Supplement: Supplementary file 1 [file Data_Sheet_1.pdf]

Supplementary Tables

Table S1. Kruskal-Wallis rank sum test for treatment effects

**Site**	**Alpha Diversity**	**chi-squared**	**df**	**p-value**
Shallow Shelf	Richness	11.44	7	0.12
	Shannon Diversity	14.33	7	**0.05**
	Pielou's evenness	13.83	7	**0.05**
Shallow Slope	Richness	5.26	7	0.63
	Shannon Diversity	14.00	7	**0.05**
	Pielou's evenness	14.45	7	**0.04**
Deep Shelf	Richness	3.71	7	0.81
	Shannon Diversity	10.20	7	0.18
	Pielou's evenness	12.44	7	0.09
Deep Slope	Richness	3.25	7	0.86
	Shannon Diversity	3.84	7	0.80
	Pielou's evenness	6.65	7	0.47

Table S2. Concentration of metals and NO_3 in the different treatments

	**Metal concentration nmol/L**	**NO_3 concentration umol/L**
**Mn**	6.02	0.10
**Fe**	11.99	0.20
**Co**	0.12	0.002
**Ni**	17.97	0.30
**Cu**	6.97	0.13
**Zn**	19.99	0.39
**Mix**	all together	1.12

Supplementary Figure legends

Figure S1. OTU Richness of heterotrophic prokaryotes in response to Co, Cu, Fe, Mn, Ni, Zn and the combination of all (Mix), and in unamended controls (Ctrl), experiments performed with water collected from (A) surface-coastal, (B) deep-coastal (C) surface-open ocean, and (D) deep-open ocean.

Figure S2. Shannon diversity index of heterotrophic prokaryotes in response to Co, Cu, Fe, Mn, Ni, Zn and the combination of all (Mix), and in unamended controls (Ctrl), experiments performed with water collected from (A) surface-coastal, (B) deep-coastal (C) surface-open ocean, and (D) deep-open ocean.

Figure S3. Relative abundance of prokaryotic 16S rRNA sequences, at the phylum level, in response to Co, Cu, Fe, Mn, Ni, Zn and the combination of all (Mix), and in unamended controls (Ctrl), experiments performed with water collected from (A) surface-coastal, (B) deep-coastal (C) surface-open ocean, and (D) deep-open ocean. Only phyla with a relative abundance ≥1% are included.

Figure S4. Relative abundance of prokaryotic 16S rRNA sequences, at the genus level, in response to Co, Cu, Fe, Mn, Ni, Zn and the combination of all (Mix), and in unamended controls (Ctrl), experiments performed with water collected from (A) surface-coastal, (B) deep-coastal (C) surface-open ocean, and (D) deep-open ocean.

Figure S5. Total abundance of reads of the 16S rRNA sequences of the genus *Thalassolituus*, in response to Co, Cu, Fe, Mn, Ni, Zn and the combination of all (Mix), and in unamended controls (Ctrl), experiments performed with water collected from surface-coastal, deep-coastal, surface-open ocean, and deep-open ocean.
