# Supplementary figures and images for: Specific Effect of Trace Metals on Marine Heterotrophic Microbial Activity and Diversity: Key Role of Iron and Zinc and Hydrocarbon-Degrading Bacteria
Source: Front Microbiol. 2018 Dec 19;9:3190. doi: 10.3389/fmicb.2018.03190 (PMC6306045; doi:10.3389/fmicb.2018.03190)

OTU Richness

Shelf

Slope

Shallow

Deep

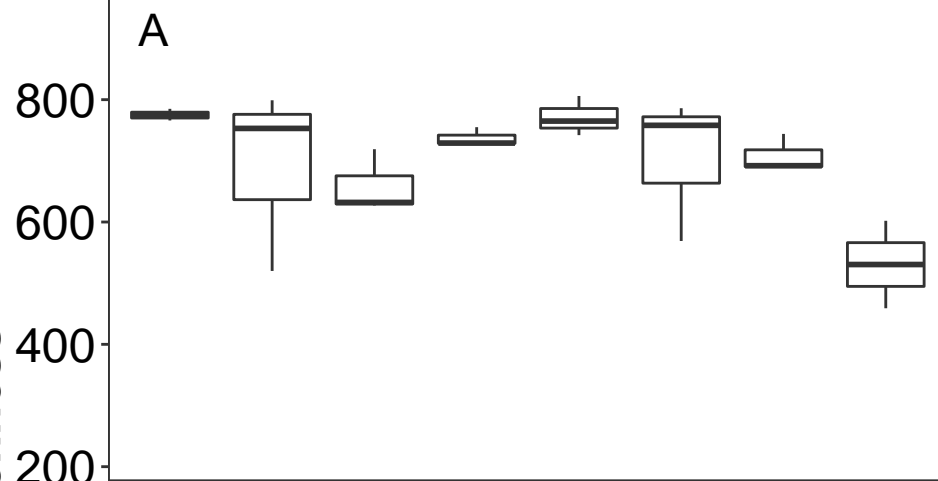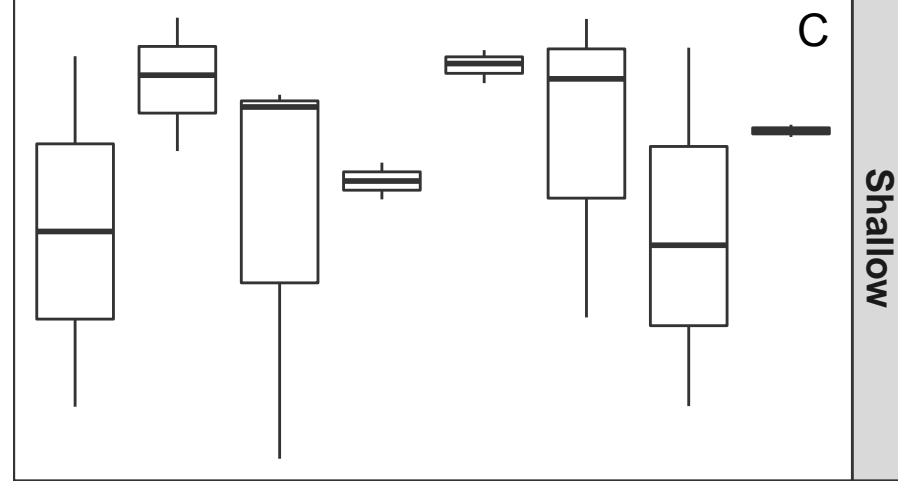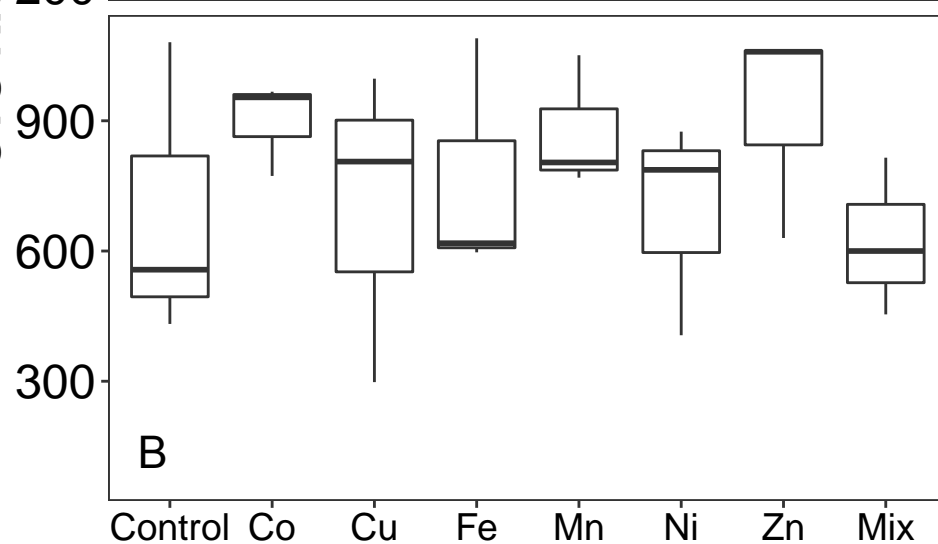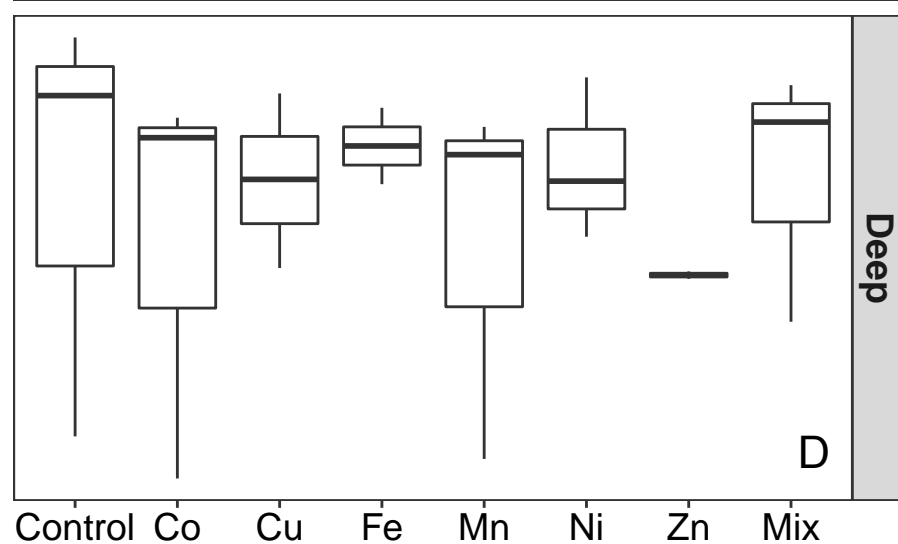

Treatment

Supplement: Supplementary file 2 [file Data_Sheet_2.pdf]

Shannon Diversity

Shelf

Slope

Shallow

Deep

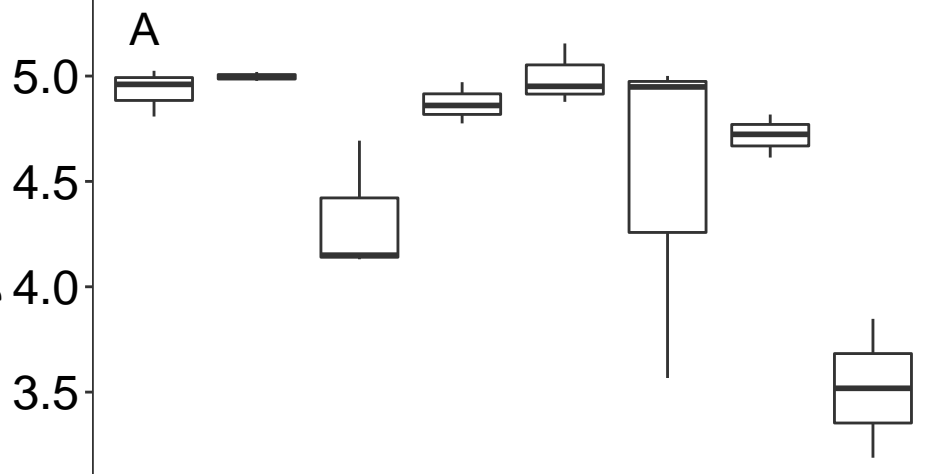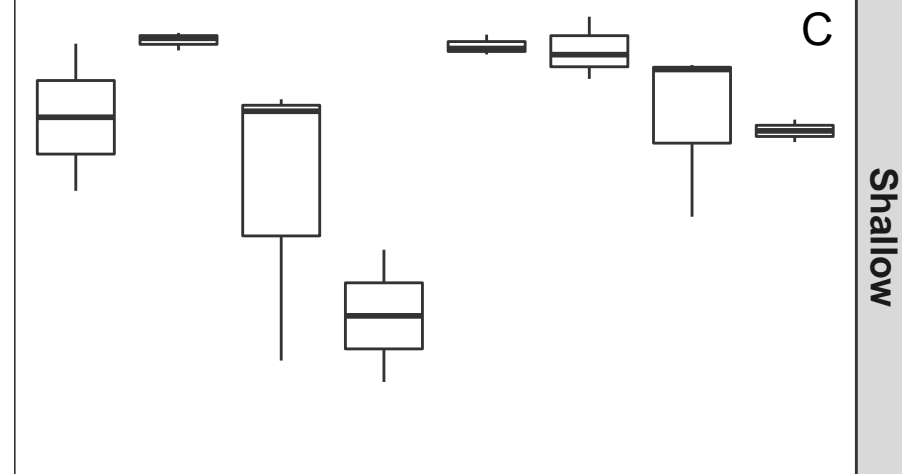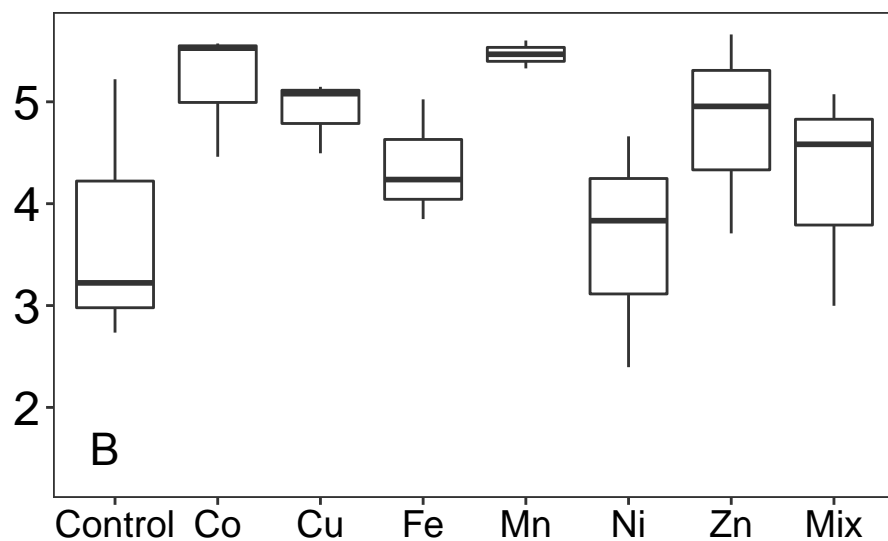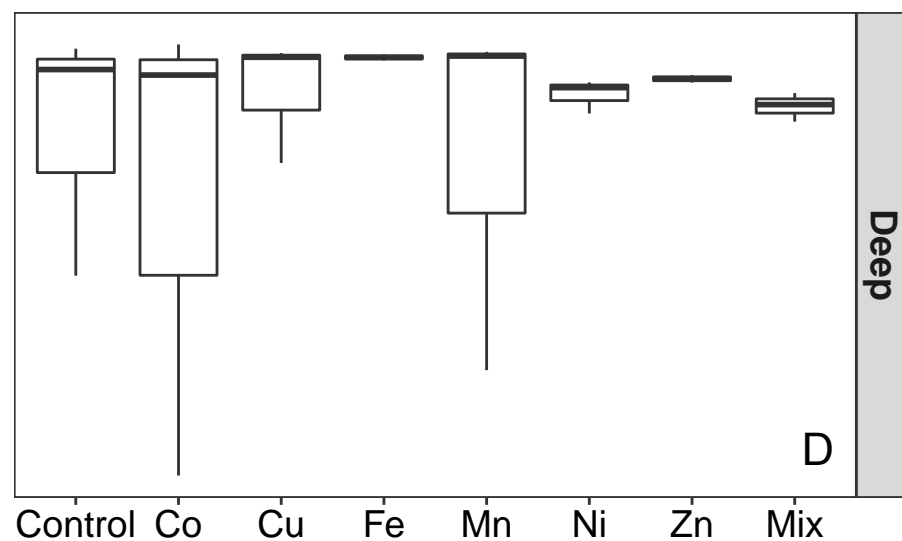

Treatment

Supplement: Supplementary file 3 [file Data_Sheet_3.pdf]

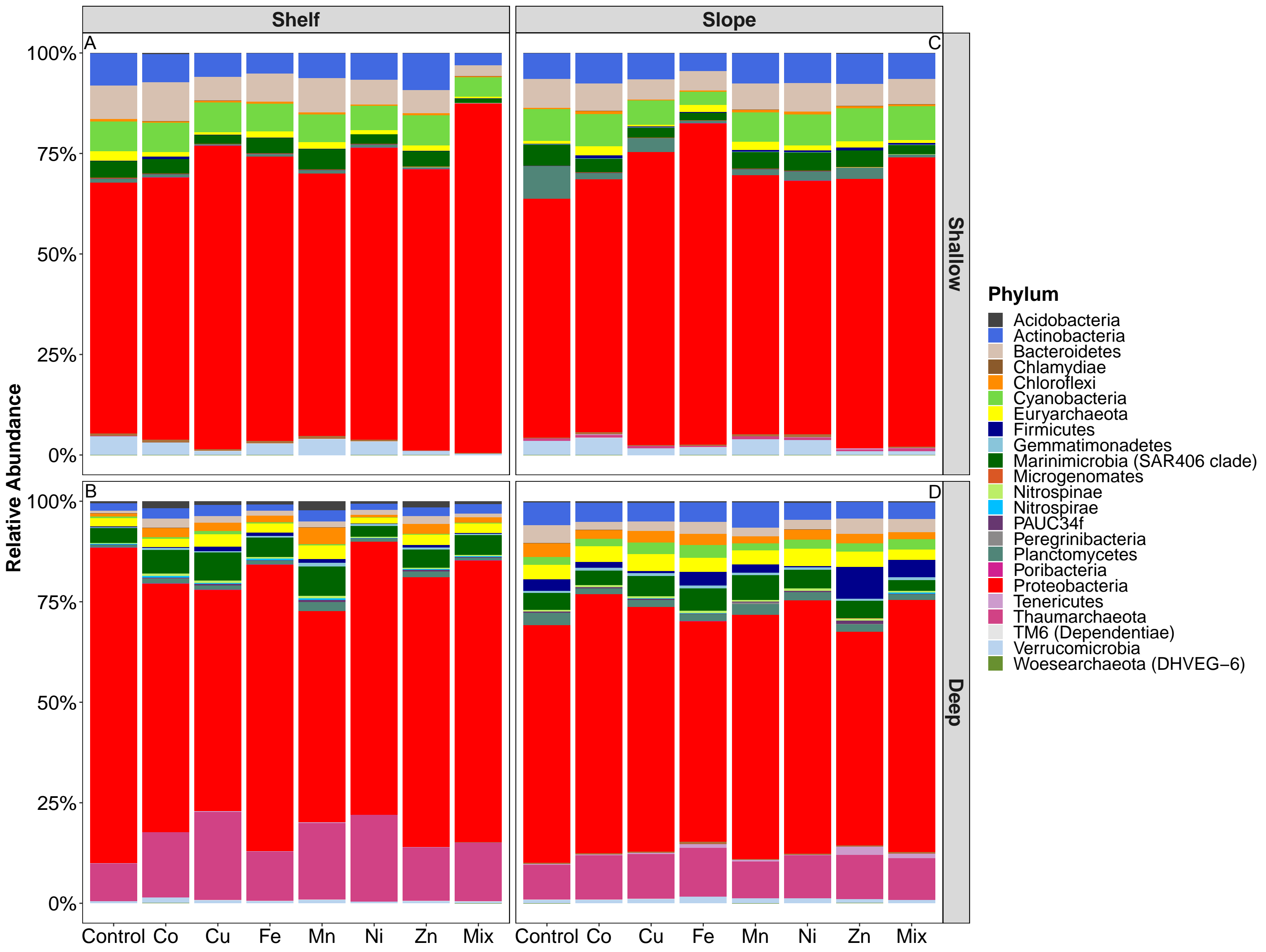

Supplement: Supplementary file 4 [file Data_Sheet_4.pdf]

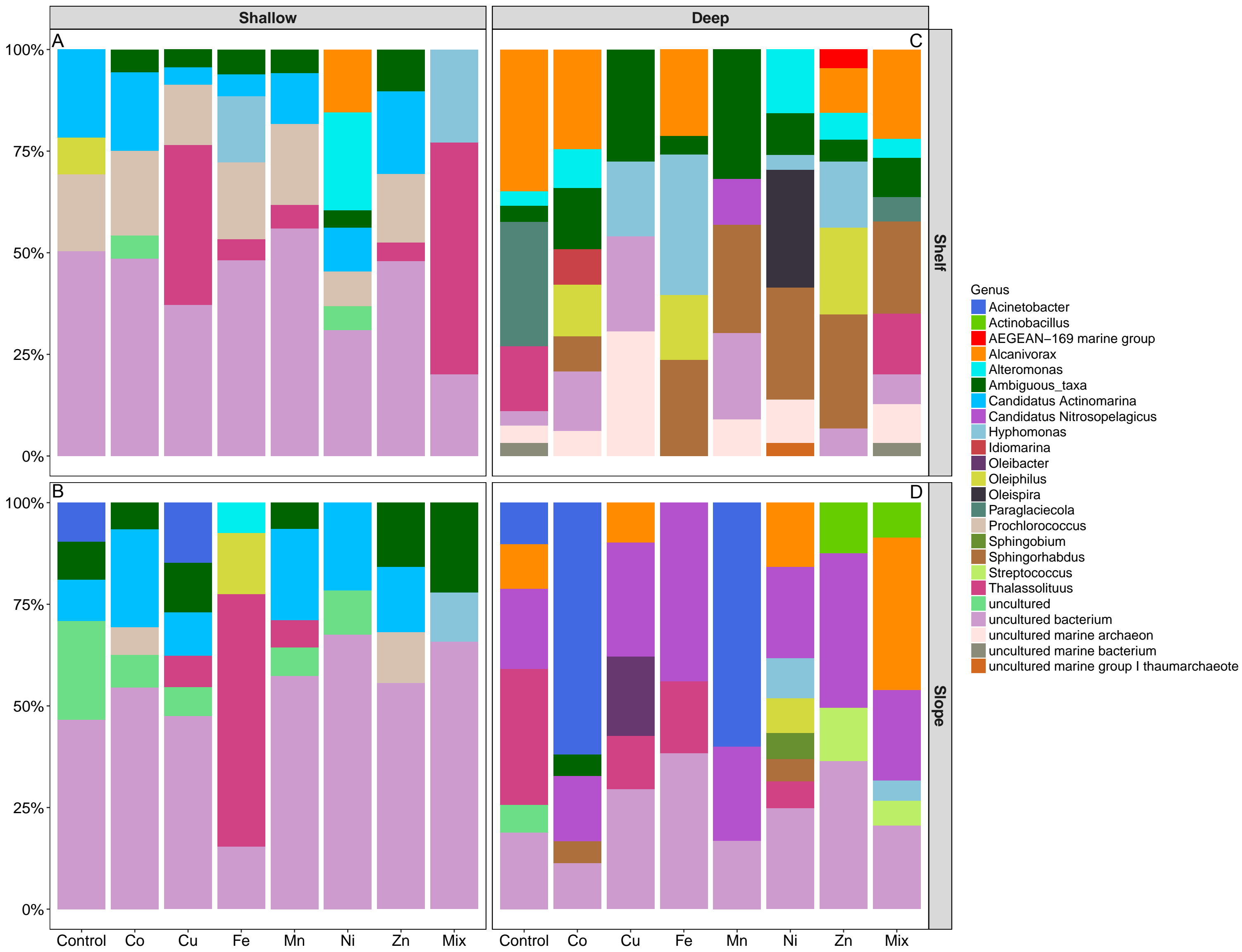

Supplement: Supplementary file 5 [file Data_Sheet_5.pdf]

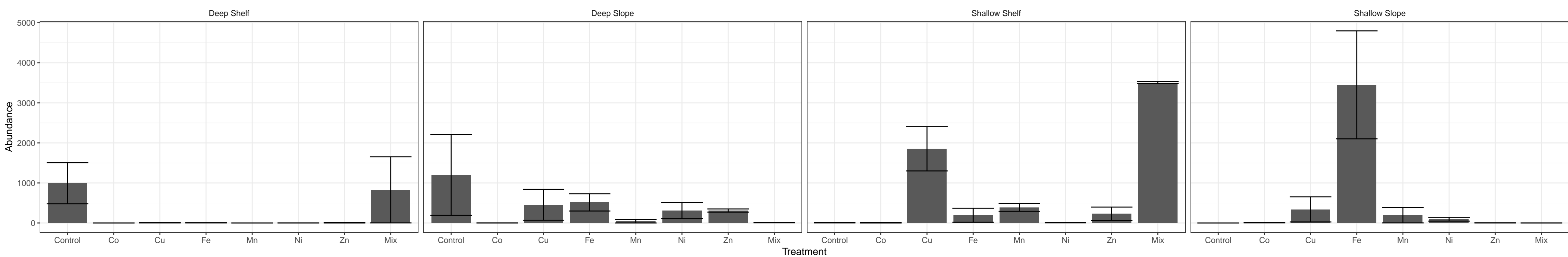

Supplement: Supplementary file 6 [file Data_Sheet_6.PDF]
